# Changes in Non-Coding RNA in Depression and Bipolar Disorder: Can They Be Used as Diagnostic or Theranostic Biomarkers?

**DOI:** 10.3390/ncrna6030033

**Published:** 2020-08-24

**Authors:** Andrew Gibbons, Suresh Sundram, Brian Dean

**Affiliations:** 1 The Florey Institute for Neuroscience and Mental Health, Parkville, The University of Melbourne, Melbourne, Victoria 3052, Australia; Suresh.Sundram@monash.edu (S.S.); brian.dean@florey.edu.au (B.D.); 2 The Department of Psychiatry, Monash University, 27-31 Wright Street, Clayton, Victoria 3168, Australia; 3 The Centre for Mental Health, Swinburne University of Technology, Hawthorn, Victoria 3122, Australia

**Keywords:** mood disorders, major depressive disorder, bipolar disorder, antidepressants, mood stabilisers, miRNA, lncRNA, Biomarkers

## Abstract

The similarities between the depressive symptoms of Major Depressive Disorders (MDD) and Bipolar Disorders (BD) suggest these disorders have some commonality in their molecular pathophysiologies, which is not apparent from the risk genes shared between MDD and BD. This is significant, given the growing literature suggesting that changes in non-coding RNA may be important in both MDD and BD, because they are causing dysfunctions in the control of biochemical pathways that are affected in both disorders. Therefore, understanding the changes in non-coding RNA in MDD and BD will lead to a better understanding of how and why these disorders develop. Furthermore, as a significant number of individuals suffering with MDD and BD do not respond to medication, identifying non-coding RNA that are altered by the drugs used to treat these disorders offer the potential to identify biomarkers that could predict medication response. Such biomarkers offer the potential to quickly identify patients who are unlikely to respond to traditional medications so clinicians can refocus treatment strategies to ensure more effective outcomes for the patient. This review will focus on the evidence supporting the involvement of non-coding RNA in MDD and BD and their potential use as biomarkers for treatment response.

## 1. Introduction

Major depressive disorders (MDD) and bipolar disorders (BD) are major psychiatric illnesses characterized by extreme and disruptive states of mood and emotional response. As such, MDD, also called unipolar depression or clinical depression, is characterised by prolonged and recurrent periods of extreme sadness and hopelessness, while the symptoms of BD, formerly known and manic depression, involve alternating episodes of depression and mania interspersed with periods of euthymia. The manic episodes are characterised by increased elation, excitability and agitation. In addition to these affective symptoms, sufferers may also experience severe cognitive deficits and psychotic symptoms, further disrupting their ability to function within society [1]. Finally, both disorders are associated with an increased risk of suicide with estimates of 31% of people with MDD and 34% of people with BD attempting suicide over their lifetime [2,3]. Exacerbating this problem, some of the drugs developed to treat these illnesses are associated with an increased risk of suicidal ideation [4].

The underlying cause of MDD and BD remain unknown; however, both genetic and environmental factors are thought to contribute to their etiologies [5,6,7,8]. MDD and BPD share obvious similarities in their depressive symptoms and can be difficult to distinguish upon first presentation to the clinic; with the diagnosis of BD often made after manic episodes present in a patient who was initially diagnosed with MDD [9]. However, familial studies report a stronger genetic association between BD and schizophrenia [10], suggesting BD may share commonalities with that disorder. This led to a hypothesis that MDD and BD are part of a continuum of overlapping, psychiatric illness risk genes that includes disorders such as autism spectrum disorder and schizophrenia [11,12].

This stronger genetic association between BD and schizophrenia compared with MDD has been seen when examining similarities in RNA expression of these three illnesses. A recent comparison study examining RNA-Seq data from several post-mortem cortical regions showed strong correlations between the transcriptomes of subjects with schizophrenia and BD with moderate correlations in gene expression between MDD and BD [13]. However, this contrasts to an expression microarray study that shows that are many more changes in gene expression in the dorsolateral prefrontal cortex from patients with MDD and BD than there are in the frontal pole and the anterior cortex [14]. Significantly, there were highly correlated changes in gene expression in the dorsolateral prefrontal cortex from patients with MDD and BD supporting the idea of a common pathophysiology. By contrast, changes in gene expression in schizophrenia are much more prevalent in the frontal pole compared to the dorsolateral prefrontal cortex and anterior cingulate [15]. These microarray data are in general agreement with a proteomic study, where BD and MDD showed a greater similarity at the protein level compared to schizophrenia [16].

The difficulty is distinguishing the diagnostic boundaries of psychiatric illness by their genetic, transcriptomic and proteomic profiles has proved problematic for identifying diagnostic and theranostic biomarkers, characteristics that can be objectively measured and evaluated as an indicator of pathologic processes or biological responses to a therapeutic intervention [17], for psychiatric illness, and its effective treatment [18]. Thus, while the field of biological psychiatry sees promise in identifying differences in gene mutations, and RNA and protein levels that can distinguish MDD and BD from their related disorders, definitive biomarkers of these disorders have yet to be employed in the routine diagnostic testing in the clinic [18,19]. The lack of cohesion between the genetic, mRNA, and protein literature in explaining the apparent symptom relationships, both within mood disorders and with other psychiatric disorders [10,13,14,16], points to the possibility that the dysregulation of non-coding RNA, as post transcriptional regulators of protein expression, may play an important role explaining how the clinical characteristics of MDD and BD manifest from their respective genetic backgrounds.

It is now known that non-coding RNA play a major role in regulating the transcription and translation of genes into proteins and there is a growing realization of the importance of this diverse group of RNA in regulating transcription, alternative splicing and translation of coding RNA in both the developing and mature brain [20]. Thus, this additional layer of control between gene expression and protein translation may be key in understanding how a genetic predisposition to psychiatric illness could eventuate into clinically definable disorders, such as MDD and BD. To date, most studies have focused on understanding how micro RNA (miRNA) contribute to developing MDD and BD with several miRNA reported to be altered in the central nervous system (CNS) and the periphery, both in subjects with MDD and BD [21,22]. However, recent evidence suggests abnormal expression of long non-coding RNA (lncRNA) also underlies the molecular dysfunction in these disorders [23,24]. Furthermore, reports that the expression levels of several miRNA in the blood are altered in response to medicating patients with psychotropic drugs offers the potential to use such changes in miRNA expression to predict a patient’s response to medication, allowing better treatment choices for the clinician [21]. We have previously reviewed the evidence supporting non-coding RNA involvement in schizophrenia [25] and found significant evidence to support the hypothesis that non-coding RNAs have a significant role in the pathophysiologies of psychiatric disorders. We now further address that hypothesis by reviewing the evidence surrounding the role of non-coding RNA in the pathophysiologies of MDD and BD.

## 2. The Role of Micro-RNA in Gene Transcription and Translation

**Figure 1:**
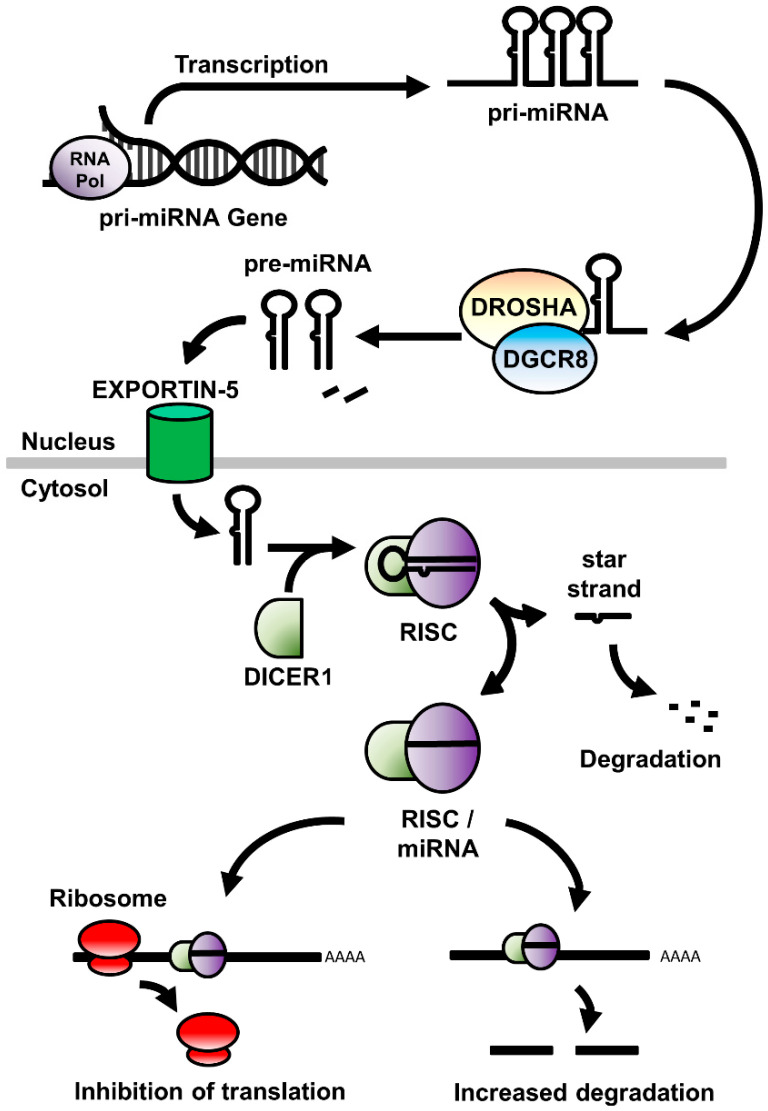
A schematic of the expression, processing, and mode of action of miRNA in the cell. RNA Pol: RNA polymerase; miRNA: micro RNA; DROSHA: Drosha Ribonuclease III; DGCR8: DiGeorge syndrome chromosomal region 8; RISC: RNA-induced silencing complex.

miRNA are a diverse family of small (20–22 nucleotides) non-coding RNA molecules that regulate the translation of mRNA by binding to complementary sequences within the transcript. As a consequence, this changes the stability or rate of degradation of the mRNA, or physically obstructs the interaction between the mRNA and the cell’s translational machinery by altering the ability of expressed mRNA to be translated into protein [26,27]. miRNA are first transcribed as a series of 1 to 6 precursor (pre-) miRNA within a larger primary (pri-) miRNA gene sequence (Figure 1). This pri-miRNA sequence is processed within the nucleus by the Drosha Ribonuclease III/DiGeorge syndrome chromosomal region 8 (DROSHA/DGCR8) enzyme complex to release the pre-miRNA, a stem-looped RNA molecule consisting of the mature miRNA sequence and its complementary (star-) strand, and is exported to the cytoplasm via the Exportin-5 nucleocytoplasmic shuttle protein. Once in the cytoplasm, the ribonuclease DICER1 cleaves the hairpin loop and loads the mature miRNA sequence within the RNA-induced Silencing Complex (RISC), a multiprotein complex that facilitates both the binding of miRNA to, and the subsequent cleavage of, the mRNA target. [28].

Due to their limited size, there is the potential for single miRNA to bind to common sequences in different mRNA and thereby regulating protein production from multiple genes [29]. As a result, they allow for a complex level of regulatory control between the initiation of gene expression and the ultimate translation of protein. It is, therefore, notable that such a complex organ as the brain shows some of the highest levels of miRNA expression compared to other organs and that miRNA have been shown to play essential roles in the development and maintenance of the central nervous system [20,30,31].

## 3. Micro RNA in Mood Disorders

The increasing understanding of the critical role of miRNA in gene transcription and translation has led to a growing interest in understanding their role in the molecular dysfunction of MDD and BD [21,22]. This interest has been heightened by several genome wide association studies (GWAS) that have reported single nucleotide polymorphisms (SNPs) in miRNA genes being associated with an altered risk for MDD and BD [32,33,34]. Of particular interest is the finding that some of the miRNA gene SNPs occur in miRNA that target several inflammation-related genes. Furthermore, SNPs in these miRNA genes were also found to be associated with an increased risk of autoimmune disorders [35]. This is significant as altered inflammatory pathways are thought to underlie both BD and MDD and several autoimmune disorders are associated with comorbid-depressive symptoms [36]. Functionally, it has been shown that chronic treatment of rats with the stress hormone corticosterone, which induces depression-like behaviours in the rats, leads to changes in miRNA that regulate inflammatory, neuro-development and plasticity pathways [37]. This suggests changes in miRNA may underlie fundamental dysregulated pathways in mood disorders. Recent studies have also highlighted the importance of extracellular vesicles, such as exosomes and microvesicles, in the CNS or in the periphery in individuals with mood disorders, most notably in BD [38,39,40]. Such vesicles can traffic aberrantly expressed miRNA between cells, effectively regulating RNA translation in nearby cells. As such, several differentially expressed miRNA have been detected in exosomes in plasma from patients with BD (refer to Table 1), and these vesicles may be useful for isolating miRNA from the periphery for use as biomarkers of diagnosis or treatment response [38,41,42].

One finding that could be most impactful on miRNA dysfunction in MDD has come from genetic studies that have reported the ‘A’ allele in the single nucleotide polymorphism (SNP) rs10144436 in the *DICER1* gene, which is essential for processing all miRNA, is associated with an increased risk of MDD [43]. As DICER1 is critical in the processing of miRNA, such a mutation could underlie the molecular changes in mood disorders by affecting multiple rather than a single ‘risk’ miRNA.

It remains to be proven that the changes in *DICER1* sequence is central to the pathophysiology of MDD however, even without proving that hypothesis, it is clear that there is a diverse array of miRNA that are reported to be present at altered levels in the CNS and the periphery in individuals with MDD or BD (refer to Table 1). Moreover, amongst these miRNA are a few miRNA that could be making an important contribution to disrupting major signalling pathways in in MDD and BD. As such, several studies have reported altered expression of miR-124 and miR-34a miRNA in both the CNS and the periphery of subjects with MDD and BD and evidence suggests they regulate biochemical and cellular pathways thought to be relevant the neurological dysfunction in mood disorders [43,44,45]. Furthermore, given the difficulties in modelling the clinical profile of mood disorders in animals, altered levels of the primate specific miRNA, miR-1202, in MDD suggest this miRNA may be important in understanding MDD as a clinical disorder of humans [46]. These miRNA will be discussed below.

**Table 1:** A summary of published studies that have reported altered levels of miRNA in patients and human-derived samples with Major Depressive Disorders (MDD) and Bipolar Disorders (BD).

Reference	Disorder	Tissue	Direction of Change	Altered miRNAs
Azevedo et al. 2016 [44]	MDD	ACC	Decrease	miR-184 and miR-34a
Belzeaux et al. 2012 [47]	MDD	Blood	Increase	miR-589, miR-579, miR-941, miR-133a, miR-494, miR-107, miR-148a, miR-652, miR-425-3p
	MDD	Blood	Decrease	miR-517b, miR-636, miR-1243, miR-381, miR-200c
Camkurt et al. 2015 [48]	MDD (First episode)	Blood	Increase	miR-451a, miR-17-5p, miR-223-3p
	MDD (First episode)	Blood	Decrease	miR-320a
Fan et al. 2014 [49]	MDD	PBMCs	Increase	miRNA-26b, miRNA-1972, miRNA-4485, miRNA-4498, miRNA-4743
Fang et al. 2018 [50]	MDD	Plasma	Increase	miR-132, miR-124
Gururajan et al. 2016 [51]	MDD (Treatment resistant)	Blood	Decrease	let-b, let-c
He et al. 2016 [52]	MDD	PBMCs	Increase	miR-124-3p
Hung et al. 2019 [53]	MDD	PBMCs	Decrease	let-7e, miR-21-5p, miR-146a, miR-155
	MDD	Monocytes	Decrease	miR-146a, miR-155
Issler et al. 2014 [54]	MDD	Blood	Decrease	miR-135a
	MDD (suicide)	Brain Stem	Decrease	miR135a
Kuang et al. 2018 [45]	MDD	serum	Increase	miRNA-34a-5p, miRNA-221-3p
	MDD	serum	Decrease	miRNA-451a
Lopez et al. 2014 [46]	MDD	Broca’s Area	Increase	miR-1202
Maffioletti et al. 2016 [55]	MDD	Blood	Increase	miR-199a-5p, miR-345-5p, miR-330-3p, miR-425-3p, miR-24-3p, miR-29c-5p
	MDD	Blood	Decrease	let-7a-5p, let-7f-5p, let-7d-5p, miR-1915-3p
Mendes-Silva et al. 2019 [56]	MDD (late)	Plasma	Decrease	miR-184
Roy et al. 2017 [57]	MDD	Serum, DLPFC	Increase	miR-124-3p
Song et al. 2015 [58]	MDD	CSF	Decrease	miR-16
		Blood	Decrease	miR-16
Wan et al. 2015 [59]	MDD	CSF	Increase	miR-34a-5p, miR-221-3p, let-7d-3p
	MDD	CSF	Decrease	miR-451a
	MDD	Serum	Increase	miR-125a-5p, miR-30a-5p, let-7d-3p, miR-34a-5p, miR-221-3p, miR-29b-3p, miR-10a-5p, miR-375, miR-155-5p, miR-33a-5p, miR-139-5p, miR-590-5p
	MDD	Serum	Decrease	miR-185-5p, miR-106b-5p, miR-15Bb-5p, miR-451a
Zhang et al. 2020 [60]	MDD	Plasma	Decrease	miRNA-134
Amoah et al. 2020 [42]	BD	Orbitofrontal cortex	Increase	miR-223, miR-330-3p, miR-1260, miR-193b-3p
Azevedo et al. 2016 [44]	BD	ACC	Decrease	miR-132, miR-133a, miR-212, miR-34a
Banach et al. 2017 [61]	BD	Leucocyte	Decrease	leucocyte miR-499, miR-708 and miR-1908
Banigan et al. 2013 [39]	BD	DLPFC exosomes	Increase	miR-29c
Bavamian et al. 2015 [62]	BD	Cerebellum	Increase	miR-34a
Camkurt et al. 2020 [63]	BD	Plasma	Increase	miR-29a-3p, miR-106b-5p, miR-107, and miR-125a-3p
	BD (manic vs. euthymic)	Plasma	Increase	miR-106a-5p and miR-107
Ceylan et al. 2020 [38]	BD	Plasma exosomes	Increase	miR-484, miR-652-3p, miR-142-3p, miR-126-3p, miR-301a-3p, miR-30b-5p, miR-15a-5p, miR-15a-5p
	BD	Plasma exosomes	Decrease	miR-185-5p, miR-25-3p, miR-92a-3p, mir-376b-3p, let-7i-5p
Choi et al. 2017 [40]	BD	ACC exosomes	Increase	miR-149
Fries et al. 2019 [41]	BD	Plasma exosomes	Increase	miR-4516, miR-29c-3p, miR-22-3p, miR-6808-5p, miR-7977, miR-142-3p, miR-1185-2-3p, miR-6791-5p, miR-3194-5p, miR-6090, miR-21-5p, miR-3135b, miR-92a-3p, miR-7975
	BD	Plasma exosomes	Decrease	miR-1281, miR-6068, miR-8060, miR-4433a-5p, miR-1268b, miR-1238-3p, miR-133a-3p, miR-188-5p, miR-6775-5p, miR-6800-3p, miR-3620-5p, miR-5739, miR-451a, miR-1227-5p, miR-7108-5p, miR-671-5p, miR-6727-5p, miR-6125, miR-6821-5p
Lee et al. 2020 [64]	BDII	Serum	Increase	miR-7-5p, miR-23b-3p, miR-142-3p, miR-221-5p, miR-370-3p
Maffioletti et al. 2016 [55]	BD	Blood	Increase	miR-140-3p, miR-30d-5p, miR-330-3p, miR-330-5p, miR-720-5p, miR-3158-3p, miR-4521-5p, miR-345-5p, miR-1973-5p, miR-378a-5p, miR-21-3p, miR-29c-5p
	BD	Blood	Decrease	miR-1915-5p, miR-1972-5p, miR-4440-5p, miR-4793-3p
Rong et al. 2011 [65]	BD (manic vs. euthymic)	Plasma	Decrease	miRNA-134
Tabano et al. 2019 [66]	BD (manic vs. control)	Plasma	Increase	miR-150-5p, miR-25-3p, miR-451a, miR-144-3p
	BD (manic vs. control)	Plasma	Decrease	miR-363-3p, miR-4454 + has-miR-7975, miR-873-3p, miR-548al, miR-598-3p, miR-4443, miR-551a, miR-6721-5p
Walker et al. 2015 [67]	BD	Blood	Increase	miRNA miR-15b, miR-132, miR-652
Wang et al. 2018 [68]	BD	Broca’s Area	Decrease	microRNA-124-3p
Zhang et al. 2020a [60]	BD	Plasma	Decrease	miRNA-134

ACC: anterior cingulate cortex; CSF: cerebrospinal fluid; DLPFC: dorsolateral prefrontal cortex; PBMC: peripheral blood mononuclear cell.

## 4. miR-124

The miRNA miR-124 has been found to be increased in serum [57], plasma [50], and peripheral blood mononuclear cells (PBMC) from patients with MDD [52] as well as the dorsolateral prefrontal cortex (DLPFC) from post-mortem subjects with MDD [57] (see Table 1). Furthermore, miR-124 levels in PBMC are shown to decrease following eight weeks of individually tailored pharmacotherapy suggesting the genes it regulates are relevant to the pathways targeted by antidepressant drugs [52]. In vivo and in vitro characterisation of target gene response shows that increased levels of miR-124 are associated with decreased levels of RNA for the glucocorticoid receptor as well as the α-Amino-3-Hydroxy-5-Methyl-4-Isoxazolepropionic Acid Receptor (AMPAR) and *N*-Methyl-d-Aspartate Receptor (NMDAR) ionotropic glutamate receptors [57]. Together, these data suggest that dysregulation of this miR-124 may play a role in the disrupted immune-related processes and glutamatergic dysfunction in seen depressive illnesses [57]. By contrast, miR-124 levels have been reported to be decreased in Broca’s Area of the frontal lobe in males with MDD and expression levels correlated with target genes in cellular stress pathways. These data suggest dysregulated expression of miR-124 may vary in specific regions of the brain in a sex-specific manner, potentially affecting different gene pathways. Agreeing with findings in the periphery and the DLPFC, chronic treatment of rats or mice with corticosterone, a stress hormone that induces a depression-like behavioural outcome in rodents, also results in the increased expression of miR-124 in the prefrontal cortex and hippocampus, which can be reduced by the selective serotonin reuptake inhibitor (SSRI), fluoxetine, [57,69]. These mice also show decreased hippocampal dendritic spine density, which can be recovered to control levels with a miR-124 antagomir [69], suggesting the increase in miR-124 is likely to impact synaptic plasticity in the brain. Furthermore, antagonism of miR-124 restores the disruption in sucrose preference and reduces immobility in the tail suspension test, behavioural models of anhedonia and defeat in depression that are seen in corticosterone-treated mice, suggesting that miR-124 acts as to disrupt molecular pathways involved in the depressive symptoms of MDD [69].

These findings in the corticosterone treated mouse contrast findings from the chronic mild stress mouse, another stress-induced model of depression, where chronic mild stress in mice was shown to reduce levels of hippocampal miR-124, with the tricyclic antidepressant imipramine restoring miR-124 levels. Overexpression of miR-124 within the hippocampus of these mice resulted in similar improvement in depression-like behaviour and increased hippocampal dendritic spine density [70]. Currently, it is not clear whether the differences between studies reflect differences in the model, differences in the genetic backgrounds of the mouse strains or differences in the administration route of the mi-RNA and mi-RNA antagonists. However, these studies taken with findings of decreased miR-124 levels in Broca’s Area in males with MDD suggest complex changes in levels of miR-124 could be contributing to the depressive symptoms of MDD. Interestingly, in both the chronic mild stress and corticosterone treated mouse studies, administration or antagonism of miR-124 in untreated, control mice had no effect on behaviour or dendritic spine density [69,70], which could mean that miR-124 could be impacting on an already disrupted molecular framework in MDD rather than directly inducing depressive symptoms.

## 5. miR-34a

The miR-34 family consists of three related miRNA encoded by two unique transcripts, one encoding miR-34a and the other encoding miR-34b and miR-34c [71]. miR-34b and miR-34c are reported to be increased in blood from first episode MDD [72] with miR-34c shown to be reduced by escitalopram treatment [73]. Contrasting the changes in miR-34b and miR-34c levels in the blood, it is the level of miR-34a that is reported to be lower in cerebrospinal fluid (CSF) [59] and the anterior cingulate [44] from subjects with MDD and BD; a CNS region thought to be functionally important in mood and emotional response [74]. Notably, the common decrease in miR-34a in MDD and BD in the anterior cingulate contrasted diagnosis specific decreases in miR-184 in MDD and miR-122, miR-132 and miR-133a in BD suggesting that while miR-34a may regulating abnormal pathways affected in mood disorders, these latter miRNA may be important in distinguishing the biochemical dysfunction in MDD from that in BD [44]. However, another study failed to detect a change in miR-34a in the anterior cingulate in MDD but did report an increase in that miRNA in their cerebellum [62]. Changes in miR-34a were only found in the cerebellum from drug naive subjects and, therefore, differences in subject drug treatment status could account of the discrepancies between in the two studies [44,62].

Functional assays of predicted miR-34a targets show that miR-34a can decrease the activity of the Nuclear Receptor Coactivator 1 (NCOA1) and the Nuclear Receptor Co-repressor 2 (NCOR2), in vitro [44]. These genes act to regulate the transcriptional machinery of the cell. Thus, it is of interest that the levels of expression of both NCOA1 and NCOR2 have been reported to be decreased in the anterior cingulate from subjects with MDD, whilst levels of expression of these genes were not altered in BD [44]. This would suggest that there are other regulatory mechanisms in place in MDD and BD that lead to substantial differences in the biochemical outcome of decreased miR-34a in the two disorders. Additionally, in immortalised neural progenitor cells derived from patients with BD, overexpressing miR-34a has been found to silence the expression of the membrane-cytoskeleton linker enzyme Ankyrin-3 (ANK3) and the calcium channel subunit Calcium Voltage-gated Channel Auxiliary Subunit β3 (CACNB3), inhibiting differentiation and dendritic development, changes that are not seen in human neural progenitor cells derived from control cases [62]. This suggests the altered expression of miR-34a may disrupt various neurodevelopmental processes that have been proposed to underlie BD [75].

## 6. miR-1202

It has become clear that not all aspects of MDD can be modelled in rodents, suggesting that there are human specific aspects to the pathophysiology of MDD. It is therefore significant that levels of miR-1202, a CNS enriched miRNA that is specific to primates, are decreased Broca’s Area from subjects with MDD [46]. By contrast, clinical studies report elevated miR-1202 in serum and whole blood from patients with MDD [46,76] (see to Table 1). The changes in miR-1202 levels in MDD is notable because one of its predicted targets is RNA encoding the metabotropic glutamate receptor 4 (GRM4) and the G-allele in the *GRM4* SNP rs2229901, which is located within the miR-1202 binding site of the *GRM4* coding sequence, is associated with an increased risk of MDD. rs2229901 is positioned outside of the sequences that directly hybridise to miR-1202 and is likely to alter the secondary structure of the miR-1202 binding site rather than reduce the sequence complementarity between miR-1202 and GRM4 [77]. The relationship between miR-1202 and GRM4 would appear to be functional, as in vitro studies have shown that treating Human Embryonic Kidney 293 (HEK293) cells with exogenous miR-1202 reduces the level of GRM4 protein levels [46]. Thus, changes in levels of miR-1202 in MDD could have a role in the disruption of the glutamate system, which has been suggested to be involved in the pathophysiology of MDD [78,79].

There may also be a role for miR-1202 in drug responsiveness, as treating human neural progenitor cells with either the tricyclic antidepressant (TCA) imipramine or the SSRI citalopram increased the levels of miR-1202 and decreased GRM4 expression [46]. This suggests miR-1202 may be involved in pathways that regulate drug-responsiveness to a number of classes of antidepressant drugs with apparent differences in pharmacologies. Adding to this argument is the finding that patients that have responded to treatment with citalopram have decreased levels of miR-1202 before treatment that increase following antidepressant drug treatment. As blood miR-1202 levels in treatment, non-responders are not significantly different from control cases, both before and after treatment, it could be that levels of blood miR-1202 may be a useful biomarker of treatment response in patients with MDD [46].

## 7. Long Non-Coding RNA in Mood Disorders

lncRNA represent the majority of the non-coding transcriptome and are characterised as non-coding transcripts over 200 nucleotides long. Functionally, they act to regulate gene transcription by affecting the recruitment of transcription factors, chromatin remodelling or alternative splicing of the transcript, or to regulate translation of transcribed mRNA by binding to complementary mRNA sequences or by sequestering miRNA that target the mRNA in order to control the stability of the mRNA [80,81,82,83,84,85,86]. Recently, several high throughput expression-profiling studies have identified differentially expressed lncRNA, both in the CNS and periphery, in individuals with MDD or BD and that some of these changes relate to comorbidities such as suicidal ideation and anxiety [23,24]. One confound in understanding the role of lncRNA in MDD and BD is that many of their targets have yet to be fully characterised.

A study measuring levels of RNA in PBMCs from patients with MDD showed large reductions in the expression of six lncRNA: LINC02151, LINC02152, NONHSAG045500, LINC02153, NONHSAT034045, and NONHSAT142707 [87]. A subsequent study confirmed the downregulation of these lncRNA in MDD but found levels of an additional three altered lncRNA (TCONS_L2_00001212, NONHSAT102891, ENST00000591189) in PMBC from subjects with MDD [88]. When these five lncRNA were included in an in silico analysis of gene interactions, it was discovered the changes in MDD would affect the transcriptional machinery of cells and would impact on regulation of neuronal growth and survival, and inflammatory processes [88]. Further analyses of the data showing changes in LINC02151, LINC02152, NONHSAG045500, LINC02153, NONHSAT034045, and NONHSAT142707 in patients with MDD showed they were only present in PMBC from subjects with MDD who had a past history of attempted suicide [89]. This finding led to the suggestion that measuring levels of these six lncRNA could be a useful biomarker for suicidal ideation in MDD.

In trying to interpret understandable changes in biological function associated with changes in levels of lncRNA, it is highly significant that overexpression of NONHSAG045500 caused a downregulation of the serotonin transporter (SERT) in SK-N-SH neuroblastoma cells. SERT is critical in acting to removing serotonin from the synapse and is also the target for the SSRI class of antidepressant drugs, which block serotonin uptake [90]. It is, therefore, feasible that in some subjects with MDD, the lower level of NONHSAG045500 has resulted in upregulation of SERT and lower levels of synaptic serotonin, which in turn contributes to the onset of MDD. This would also suggest that the mechanism of action of the SSRIs is to normalise the increased uptake of serotonin by the higher levels of SERT caused by the decreased levels of NONHSAG045500. Therefore, a better understanding of the impact of changes in lncRNA, such as NONHSAG045500, could give new insights as to potential mechanisms that could be targeted by drugs to relieve the symptoms of MDD.

The expression of another of these MDD associated lncRNA, LINC02151, is also reduced in the hippocampus of the chronic mild stress mouse, with expression increasing to control levels following the administration of imipramine. Over expression of LINC02151 in these mice upregulates phosphorylated-GSK3β (p-GSK3β) protein and β-catenin in the hippocampus suggesting the deficits in LINC02151 seen in humans may involve disruption of the Wnt/β-catenin pathway, which has previously been implicated in the molecular dysfunction of mood disorders [91]. However, in examining the behavioural response of chronic mild stress mice to LINC02151 overexpression, LINC02151 was found to ameliorate the anxiety-like behaviour normally seen in this model, such as reduced social interaction and increased latency to feeding, but did not affect the defeat-like behaviour of increased immobility in the forced swim test [92]. This may suggest that the effects of decreased LINC02151 in patients with MDD [87] may be relevant to the co-morbid stress and anxiety that is associated with depression. Supporting this idea, a comparative analysis of lncRNA expression in patients with other psychiatric illnesses showed that LINC02151 was amongst several lncRNA, which also included LINC02152, LINC02153, NONHSAG045500, NONHSAT034045, NONHSAT142707, that were decreased in PBMCs from patients with MDD and from patients with anxiety disorders [93]. Therefore, decreased levels of these lncRNA could be useful in identifying individuals at risk of comorbid anxiety in MDD, which is associated with poorer prognosis [94]. Interestingly, contrasting the decreased expression seen in MDD and anxiety disorders, the expression level of these lncRNA in schizophrenia is elevated compared to control levels [95], which may provide some evidence that the non-coding transcriptome plays an important role in orchestrating the abnormal expression of psychiatric illness-related genes into a clinically defined illness.

Recently lncRNA have also been implicated in sex differences in MDD with a study reporting sex-specific changes in the intergenic lncRNA LINC00473 in the CNS of subjects with MDD [96]. LINC00473 is a primate specific lncRNA with studies in carcinoma cells suggesting it acts by regulating miRNA [97,98,99]. Issler et al., measured levels of LINC00473 RNA in the post-mortem ventromedial, dorsolateral orbitofrontal, anterior cingulate and insular cortices and the subcortical regions of the ventral subiculum and nucleus accumbens of male and female subjects with MDD. Reduced LINC00473 expression was detected in all regions accept the nucleus accumbens in females but not males with MDD compared to control cases [96]. Such deficits in LINC00473 may account for some of the sex differences in symptom profile, severity and comorbidities in depressive illnesses [100,101]. Interestingly, knockdown of LINC00473 expression in neural progenitor cells had a greater effect on gene expression in female-derived cells compared to males-derived cells supporting a role in sex-specific post-transcriptional regulation. Furthermore, overexpression of LINC00473 in the chronic social defeat stress and chronic mild stress mouse models led to improved resilience to anxiety and depression-like behaviours in female mice [96], suggesting that, although primate specific, LINC00473 targets evolutionarily conserved pathways involved in depressive behaviour.

A number of studies have reported changed levels of lncRNA in patients with BD [102,103,104]. Amongst these lncRNA, several have been identified that target pathways involved in inflammation-related processes. IFNG-AS1 is an interferon Gamma (INFG) antisense lncRNA within the *INFG* locus that is lower in PBMC from patients with BD compared to controls. It is notable that this lncRNA controls the expression of the cytokine IFNG and the decreased expression of IFNG-AS1 in BD correlated with a decreasing IFNG expression and with a corresponding decrease in the levels of the pro-inflammatory cytokine IL1B [103], suggesting a functional impact of changed IFNG-AS1 levels. Similarly, Promoter of CDKN1A Antisense DNA Damage Activated RNA (PANDA), a DNA-damage activated lncRNA is also increased in blood from patients with BD [102]. The expression of PANDA is induced by the apoptotic pathway protein P53, which regulates, and is regulated by, inflammatory pathways [105]. This same study also reported increased levels of the lncRNA Taurine Up-Regulated 1 (TUG1) [102], a competing endogenous RNA that acts as a molecular sponge for the inflammation associated miRNA, miR-29b [106,107]. Whilst these findings are in peripheral tissue, changes inflammation-related lncRNA are also seen in the CNS of subjects with BD. A recent RNA-Seq study also reported increased levels of the lncRNA Lnc-PLA2G12A-1 in the post-mortem, medial frontal gyrus from subjects with BD [104]. The Lnc-PLA2G12A-1 gene is located within the *PLA2G12A* gene and likely targets the transcript of PLA2G12A, a phospholipase A2 enzyme involved in the synthesis of arachidonic acid, providing a substrate for producing the prostaglandin mediators of inflammatory response. Notably, this pathway is reported to be modulated by mood stabilisers [108] and is targeted by nonsteroidal anti-inflammatory drugs, such as celecoxib, which have been used as adjunct treatment for BD [109].

Beyond inflammation, lncRNA that target the schizophrenia candidate gene Disrupted in Schizophrenia (*DISC1*) have also been reported in BD. While it has been intensely studied for its potential role in schizophrenia [110], decreased levels of DISC1 expression have also been reported in PBMCs from patients with BD compared to controls. Notably these changes in DISC1 expression were correlated with the increased expression of DISC2, a DISC1 antisense lncRNA located within the *DISC1* locus [111]. Further implicating a role lncRNA regulated DISC1 pathways in BD is the finding that lncRNA LERFS is lower in the post-mortem frontal medial cortex in from subjects with the disorder. This lncRNA has downstream interactions with DISC1 via the negative regulation of its target, Synaptotagmin Binding Cytoplasmic RNA Interacting Protein (SYNCRIP) and, thus, different non-coding RNA regulatory mechanisms may be acting in the CNS and blood to affect the same pathways [112].

## 8. Challenges in Using Non-Coding RNA as Diagnostic Biomarkers

A major goal to psychiatric research is to identify biomarkers that will allow early and reliable diagnosis of psychiatric illness and guide their effective clinical treatment [113]. While there is strong evidence supporting the aberrant expression of miRNA in MDD and BD no single miRNA has been consistently shown to predict a diagnosis with either disorder (refer to Table 1). This, in part may reflect the symptom heterogeneity that exists within MDD and BD [114,115] and, thus, it may be necessary to define diagnostic subtypes within these disorders by their molecular profile. There is some evidence for the validity of that approach as Lei et al. identified a panel of six differentially expressed miRNA in blood: miR-7-5p, miR-23b-3p, miR-142-3p, miR-221-5p, and miR-370-3p, that differentiated patients with bipolar II disorder from healthy controls [64]. However, further studies are needed to see whether these miRNA are diagnostically specific to BDII compared to other bipolar disorders or other psychiatric illnesses. Furthermore, differences in the clinical samples used in the literature confound the identification of potential diagnostic biomarkers as several studies report differences in the miRNA profile of blood samples between using plasma, serum or whole blood [116,117] while the platforms used to measure non-coding RNA also introduce variations in their measurements [118].

At least two studies have reported changes in miRNA levels in CSF from patients with MDD [58,59]. The CSF does have several advantages as a biofluid for the detecting non-coding RNA biomarkers. When compared to blood, the ability of the blood-brain barrier to reduce the influence of other organs on gene expression in the CSF means that CSF samples should more closely reflect the changes in the non-coding transcriptome that are occurring in the brains of patients with MDD and BD. Thus, while RNAseq analysis of non-coding RNA suggests a reasonably high correlation between the miRNA transcriptomes of blood-derived biofluids and the CSF in healthy individuals, the levels of individual miRNA can differ markedly between different biofluid types [119]. This is further supported studies examining in miRNA expression in CSF and blood in patients with neurological and psychiatric disorders, where changes in diagnosis-related miRNA in the CSF do not readily correlate to those seen in blood samples [59,120,121]. Additionally, a meta-analysis of cancer-related miRNA data suggest the CSF allows both higher sensitivity and higher specificity in detecting miRNA when compared to blood [122].

However, despite the greater ability the detect pathologically relevant changes in non-coding RNA in the CSF, blood-based biofluids are generally seen as preferable for biomarker detection as whole blood, plasma and serum samples can be collected more easily, and at a lower cost with a lower risk of clinical complications. Furthermore, the potential for blood contamination of CSF samples to affect the reliable measurement of non-coding RNA means that greater diligence is needed when collecting and preparing samples measuring non-coding RNA biomarkers [123]. While CSF is often seen as a viable biofluid for biomarker discovery in disorders, such as neurodegenerative diseases [124], where CSF sampling via lumbar puncture is often performed during diagnosis, the invasiveness of CSF collection makes CSF less desirable for use in psychiatric diagnosis, where such tests are not routine and the patient’s mental well-being must be considered when performing such procedures, particularly where repeated monitoring of non-coding RNA biomarkers is required to assess treatment response.

A further consideration in the feasibility of using non-coding RNA as diagnostic biomarkers is that the disparities between the noncoding RNA reported to be altered in MDD and BD (see Table 1) may also be affected by differences in medication histories between the cohorts. Indeed, the ability of psychotropic drugs to affect non-coding RNA levels presents challenges in their use as diagnostic biomarkers. Thus, it may be that this dynamic nature of non-coding RNA expression to respond to change may make them most advantageous as predictors of treatment response enabling the more effective selection of medication for patients [125].

## 9. Non-Coding RNA as Biomarkers of Drug Response

As already suggested, there is preliminary evidence to suggest that changes in levels of non-coding RNA in the blood of patients with BD and MDD could have value as biomarkers for diagnosis or drug responsiveness [126]. For example, the use of altered plasma miRNA levels as biomarkers to predict the increased risk of suicidal ideation is currently in a Phase 2 clinical trial (clinicaltrials.gov identifier: NCT02418195) whilst a second aim of this trial is to show there levels of miRNA can be used as biomarkers of responsiveness to the fast-acting antidepressant ketamine. A significant proportion of patients with BD and MDD are refractory to antidepressant and mood stabiliser treatment, leading to poor patient outcomes caused by prescribing ineffective drugs [127,128]. Several studies have shown that the expression of many non-coding RNA, particularly miRNA, change following mood stabiliser or antidepressant treatment (see Table 2) and some data suggest such changes in miRNA levels may be predictive of non-responsiveness to medication.

Lithium and valproic acid have been commonly used for several decades to stabilise the manic and depressive symptoms of BD; however, it is still unclear how either drug achieves this effect [138,139]. Both drugs have been shown to have profound effects on the non-coding transcriptome. Notably, treating HEK-293 cells with valproic acid increases the proteasomal degradation of DICER1 leading to a reduction in miRNA processing [140]. Similarly, lithium has been shown to affect the expression of let-7e [132], which is thought to target DICER mRNA [29]. Therefore, a potential mode of action for valproate and lithium may be to reset the aberrant miRNA transcriptome that is seen in BD. In vitro studies also show that lithium has some of its most profound effects on non-miRNA, non-coding RNAs with small nuclear RNA and small nucleolar RNA levels substantially increasing in the neuroblastoma cell line SK-N-SH following lithium treatment compared to the overall levels of other non-coding and coding RNAs [141]. Focusing on lithium, a study has compared treating lymphoblastoid cells derived from lithium responder and non-responder BD patients with lithium for 7 days. Lithium treatment alters levels of over 50 miRNA, with the most significant changes seen in elevated miR-148a miR-22 and reduced miR-320a miR-125a miR-574 miR-1273h [135]. Another study has shown that treatment time is also a significant variable with acute and chronic treatment with lithium causing different changes in mi-RNA (see Chen et al. 2009 in Table 2) [129]. The effect of treatment was such a variable that only one miRNA (miR-155) had changed levels (higher) after acute (4 days) and chronic (16 days) treatment. It is also notable that changes in miR-155 levels in response to treatment also only occurred in lithium responder-derived cell lines [129,135] suggesting it may be critical in the mechanisms of action of that drug. These data suggest that a better understanding of the impact of lithium and valproate treatments on levels of non-coding RNA may help identify ways to more specifically target pathways to give therapeutic benefits to those with mood disorders.

SSRI are a commonly prescribed class of antidepressant drugs that share a common pharmacological mechanism of blocking the removal of serotonin from the synapse by SERT to increase the availability of serotonin to the serotonin receptors [142]. Several sets of potential miRNA biomarkers have been identified that are either increased or decreased in the blood following treatment with the SSRIs escitalopram [50,73], paroxetine, [45] and fluoxetine [143] (see Table 2) with evidence showing that many of the changes in miRNA levels correspond to improvements in the patient’s depressive symptoms [45]. Notably, a recent study examined miRNA changes in patients that had been prescribed SSRIs, serotonin–norepinephrine reuptake inhibitors and atypical antidepressants and it was shown that miRNA let-7e, miR-146a and miR-155 were low in PBMCs from patients with MDD and, subsequently, increased in response to antidepressant treatment. Two of these miRNA, let-7e and miR-155, were significantly altered following treatment in treatment responders, but not in non-responders [53]. Thus, miRNA may be useful as biomarkers to screen patients for general antidepressant response.

## 10. Concluding Remarks

The regulation of gene expression in both BD and MDD are characterised by complex changes in non-coding RNA expression that involve both common and distinct sets of differentially expressed RNA. This complexity in the non-coding transcriptomes of BD and MDD may explain the similarities between both the proteomes and the symptoms associated with BD and MDD given the modest genetic similarities between the disorders [16,144]. However, it is likely that they also reflect the heterogeneity in both the molecular biology and the severity and penetrance of symptoms that exists within each disorder [145]. The last decade has seen a growing interest in characterising the non-coding RNA changes that underlie BD and MDD and considerable in roads have been made into identifying differentially expressed non-coding RNA that could be used both diagnostically and to predict treatment outcomes [126]. However, the genes and pathways many of these non-coding RNA control remain to be fully characterised.

Within the CNS, changes in non-coding RNA in subjects with MDD and BD appear to vary across brain regions, which parallel the regionally discrete changes seen in coding RNA in these disorders [14]. While these diverse changes in the non-coding transcriptome across the brain speak to the functional differences of different brain regions, few studies to date have profiled MDD- and BD-associated changes in non-coding RNA across multiple brain regions within the same cohort to determine whether these changes actually reflect regional differences or differences between the cohorts used. How, or even whether such changes in non-coding RNA the CNS, relate to detectable changes in non-coding RNA in the blood remains to be elucidated.

Future efforts to understand the reasons for aberrant non-coding RNA expression in mood disorders, and the biochemical consequences of these changes, will lead to significant improvements in, not only understanding how these disorders develop, but also in understanding how we progress from potentially identifying patients with mood disorders based in treatment response to identifying effective pharmacological targets, for patients with mood disorders who are predicted to be refractory to treatment.

## Figures and Tables

**Table 2:** A summary of published studies reporting altered levels of miRNA in response to antidepressant and mood stabiliser medication in patients and human-derived samples.

Reference	Drug	Tissue	Direction of Change	miRNAs
Belzeaux et al. 2012 [47]	Various AD (8 weeks)	PBMCs	Increase	miR-20b-3p, miR-433, miR-409-3p, miR-410, miR-485-3p, miR-133a, miR-145
	Various AD (8 weeks)	PBMCs	Decrease	miR-331-5p
Bocchio-Chiavetto et al. 2013 [73]	Escitalopram (12 weeks)	Blood	Increase	miR-130b, miR-505, miR-29b-2, miR-26b, miR-22, miR-26a, miR-664, miR-494, let-7d, let-7g, let-7f, miR-629, miR-106b, let-7e, miR-103, miR-191, miR-128, miR-502-3p, miR-374b, miR-132, miR-30d, miR-500, miR-589, miR-183, miR-574-3p, miR-140-3p, miR-335, miR-361-5p
	Escitalopram (12 weeks)	Blood	Decrease	miR-34c-5p and miR-770-5p
Chen et al. 2009 [129]	Lithium (4 days) (16 days)	Lymphoblastoid cells	Increase	miR-221, miR-152, miR-15a, miR-155, miR-181c, miR-34a miR-221, miR-152, miR-155 and miR-34a
	Lithium (4 days)	Lymphoblastoid cells	Decrease	miR-494
Creson et al. 2011 [130]	Lithium (5 weeks)	Rat frontal cortex	Decrease	let-7b
Croce et al. 2014 [131]	Lithium + Valproate (48 h)	SH-SY5Y neuroblastoma cells	Decrease	miR-30a-5p
Fang et al. 2018 [50]	Citalopram (8 weeks)	Plasma	Decrease	miR-132
	Citalopram (8 weeks)	Plasma	Increase	miR-124
Hung et al. 2019 [53]	Various (4 weeks)	PBMCs	Increase	let-7e, miR-223, miR-146a, miR-155
	Various (4 weeks)	Monocytes	Increase	let-7e, miR-21-5p, miR-145, miR-146a, miR-155
Hunsberger et al. 2015 [132]	Lithium (7 days)	BD-derived lymphoblastoid cells	Decrease	let-7
Issler et al. 2014 [54]	Imipramine (3 weeks)	Mouse brain stem	Increase	miR-135a
	Fluoxetine (3 weeks)	Mouse brain stem	Increase	miR-135a
Kim et al. 2016 [133]	Valproate (7 days)	BD derived neuroprogenitor cells	Decrease	miR-1908-5p
	Valproate (7 days)	Control derived neuroprogenitor cells	Increase	miR-1908-5p
Kuang et al. 2018 [45]	Paroxetine (8 weeks)	serum	Increase	miRNA-451a
	Paroxetine (8 weeks)	serum	Decrease	miRNA-34a-5p, miRNA-221-3p
Lim et al. 2016 [134]	Asenapine (12 weeks)	Blood	Increase	miR-18a-5p, miR-19b-3p, miR-145-5p, miR-27a-3p, miR-148b-3p, miR-210-3p, miR-17-3p, miR-30b-5p, miR-106b-5p, miR-339-5p, miR-106a-5p, miR-20a-5p, miR-17-5p, miR-15a-5p
	Asenapine (12 weeks)	Blood	Decrease	miR-92b-5p, miR-1343-5p
Pisanu et al. 2019 [135]	Lithium (7 days)	Li Responder derived lymphoblastoid cells	Increase	miR-320a
	Lithium (7 days)	Li Responder derived lymphoblastoid cells	Decrease	miR-155-3p
Rong et al. 2011 [65]	Lithium	Plasma	Increase	miRNA-134
Squassina et al. 2020 [136]	Lithium (7 days)	BD derived lymphoblastoid cells	Decrease	miR-186-5p, miR-423-5p
Zhou et al. 2009 [137]	Lithium (4 weeks)	Rat hippocampus	Decrease	let-7b, let-7c, miR-128a, miR-30c, miR-34a, miR-22, miR24a
	Lithium (4 weeks)	Rat hippocampus	Increase	miR144
	Valproate (4 weeks)	Rat hippocampus	Decrease	let-7b, let-7c, miR-128a, miR-30c, miR-34a, miR-22
	Valproate (4 weeks)	Rat hippocampus	Increase	miR144

AD: antidepressant; BD: bipolar disorder; Li Responder: lithium responder; PBMC: peripheral blood mononuclear cell.
